# Self-Adaption Matched Filter and Bi-Directional Difference Method for Moving Target Detection

**DOI:** 10.3390/s18103177

**Published:** 2018-09-20

**Authors:** Shitao Zhu, Xiaoming Chen, Xuehan Pan, Xiaoli Dong, Hongyu Shi, Anxue Zhang, Zhuo Xu

**Affiliations:** 1 School of Electronic and Information Engineering, Xi’an Jiaotong University, Xi’an 710049, China; xiaoming.chen@xjtu.edu.cn (X.C.); pan.xuehan@stu.xjtu.edu.cn (X.P.); hongyushi@xjtu.edu.cn (H.S.); anxuezhang@xjtu.edu.cn (A.Z.); 2 School of Telecommunication and Information Engineering, Xi’an University of Posts and Telecommunications, Xi’an 710121, China

**Keywords:** self-adaption matched filter, low signal-to-interference-noise ratio (SINR), linear frequency modulation (LFM) signal, target detection

## Abstract

In this paper, a self-adaption matched filter (SMF) and bi-directional difference techniques are proposed to detect a small moving target in urban environments. Firstly, the SMF technique is proposed to improve the signal-to-interference-noise ratio (SINR) by using the power factor. The properties of the transmitting signal, the target echoes and the interference and noise are considered during the power factor generation. The amplitude coherent accumulation technique that extracts the coherent amplitude information of echoes after being processed by the SMF, is used to improve the SINR based on multiple measurements. Finally, the bi-directional difference technique is proposed to distinguish the target echoes and the interference/noise. Simulations and experiments are conducted to validate and demonstrate that small moving targets can be detected with high probability using the proposed method in urban environments, even with just one measurement.

## 1. Introduction

**Figure 1:**
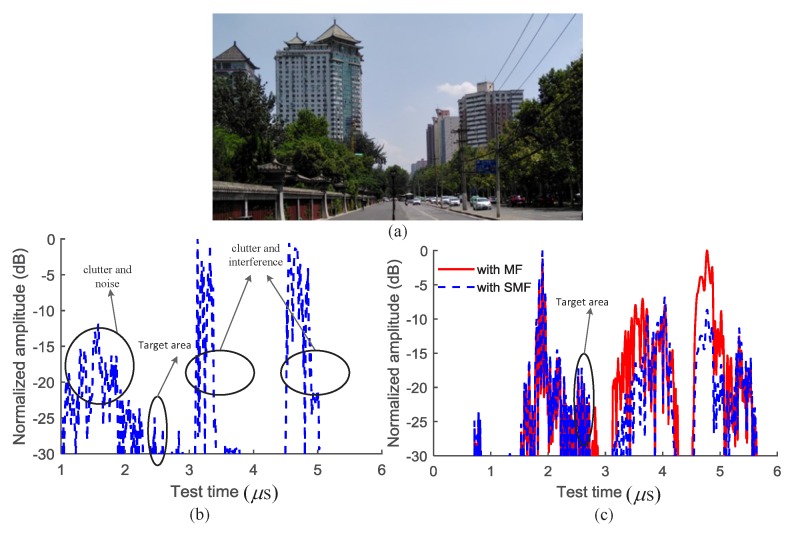
The test echo signal in the typical urban environment. (**a**) A picture of the typical urban environment; (**b**) the raw echo signal; and (**c**) The echo signal with the matched filter MF and the self-adaption matched filter (SMF).

The linear frequency modulation (LFM) signal (chirp signal) is widely used for target detection in numerous applications. The velocity and the acceleration of the target can be estimated by modeling the high-quality return signals [1,2]. The LFM signal is usually used to improve the signal-to-noise ratio (SNR) of the echoes when the information related to velocity is weak or uncontrollable [3]. When the maneuvering target is in an urban environment, the adjacent echoes overlap and the time delay between them varies. The target must be detected and distinguished from those containing several overlapped echoes with uncontrollable characteristics. It is challenging to distinguish the target using a single measurement in an urban environment (one typical scenario of which is shown in Figure 1). In target detection processing, several high time and frequency resolution analysis methods and SNR improvement methods [4,5,6,7,8,9,10,11] have been investigated.

Several studies have focused on time-frequency (TF) analysis methods [4,5,6,7,8] for the LFM signal, such as the Wigner–Ville distribution (WVD), short-time Fourier transform (STFT), and wavelet transform (WT). The merits of each TF analysis method are well-known. The high resolutions in time and frequency domains are hardly obtained simultaneously for the STFT and the WT analysis methods [6,8]. The WVD method is more effective in the single-component LFM signal analysis due to its excellent energy localization and noninterfering time and frequency resolution [4,9]. However, a cross-term appears in the multi-component LFM signal analysis [5]. The pseudo WVD method and the smooth pseudo WVD method have been presented to reduce the interference terms through the kernel function or the window function at the expense of resolution degradation [10,11,12]. The decomposition methods [13,14] are used to decompose the signal before the TF analysis. In order to further reduce the interference, feature extraction techniques [15,16] are applied to WVD image. The TF analysis methods are effective when the SINR of the echo is high [5,17,18]. However, the SINR in practical applications is usually poor, especially in the urban environment.

Target detection is difficult in the urban environment, because of the strong clutter, noise and interference [19]. Hence, the new imaging method [20], the new detection processing method [21,22], and the Multimodal Data Fusion method [23] were applied to target urban environments. In order to achieve long distance target detection, the microwave frequency should be used [1,4,14,24]. Furthermore, the high range resolution, i.e., the wideband detection system, is also required to improve the time resolution of the detection.

Due to the frequency response error and the instabilities, the wideband signal generator and the transmitting system should be corrected in order to ensure the performance of the matched filter (MF) [25,26]. The calibration can be performed using the digital method based on the priori information obtained from the auxiliary detections [25]. In addition, the time side lobe (TSL) of the target echoes after the MF processing should be restrained using the digital method in order to reduce the mutual interference of small targets. Recently, the cubic phase function (CPF) has been investigated based on the multi-component LFM [24,27,28], which presents a method of using the coherent echoes after the MF processing. Despite the above-mentioned techniques, the detection of small moving targets in urban environments is still a challenging task. The difference between the target echo and the interference should be further investigated based on the feature analysis method and the wideband detection system.

In this paper, a self-adaption moving target detection (SAMTD) method based on the single-component LFM signal is proposed. In this paper, the proposed method mainly aims to detect the unmanned aerial vehicle (the velocity is usually lower than 40 m/s.) using the pulse radar in the urban environments. The proposed method can be extended to detect the vehicle in the urban environment through adjusting the bandwidth of the transmitting signal. The signals captured by the radar are processed jointly to detect the target. Firstly, the self-adaption match filter (SMF) method is proposed to improve the SINR of the target echoes during the matched filtering when the target echo is away from the strong interference or noise. The performance of the SMF is the same as the traditional match filter when the target echoes are close to the strong interference. Then, the amplitude coherence accumulation (ACA) method is used to enhance the differences between the target echo and the interference using the multi-measurement data after removing the background noise and the coherent interference of the echo. Finally, after the SMF and the ACA processing, the bi-directional difference (BDD) of the echo is proposed to distinguish the target echoes and the interference using the difference information. The proposed method can detect the target with high probability based on multiple tests in the urban environment.

The rest of this paper is organized as follows. In Section 2, the problem formulation of detecting a target in urban environments is presented. In Section 3, the SAM filter technique, the ACA method and the BDB technique are proposed. In Section 4, the proposed method is validated through simulations and experiments. Finally, Section 5 concludes this paper.

In this paper, interference is defined as the background echo and clutter. The SINR is the ratio between the target echo and the interference (including the interference and the noise), while the SNR is the ratio between the target echo and the noise. The SINR and the SNR are calculated using the simulation data in simulations. The SINR and the SNR are obtained using the post-processing method, that is, the SINR and the SNR were obtained through comparison to standard values. The standard values are marked through measurements.

## 2. Problem Formulation of Target Detection

The baseband form of the single-component LFM transmitting signal and the matched filter are given by:

(1) $$S\left(t\right)=\mathrm{rect}\left(\hat{t}/T_{p}\right)e^{j\pi \beta {\hat{t}}^{2}},$$

(2) $$F_{m}\left(t\right)=\mathrm{rect}\left(t/T_{p}\right)e^{-j\pi \beta t^{2}},$$

where β is the chirp rate; $\hat{t}=t-mT$ with *m* and *T* being the index of the transmitting pulse and the pulse repetition time (PRT), respectively; $T_{p}$ is the pulse duration; and $\mathrm{rect}\left(\hat{t}/T_{p}\right)$ in (1) is a time rectangular window used to restrict the effective detection time interval.

In the urban detection environment, the echo signal can be described as:

(3) $$S_{r}\left(t\right)=\sum_{i}A_{i}e^{j\varphi_{i}}S_{i}\left(t-\tau_{i}\right)+N\left(t\right)$$

where $S_{i}\left(t-\tau_{i}\right)$ is the echo signal from the target (or the background) with the time delay of $\tau_{i}$; $A_{i}$ and $\varphi_{i}$ are the amplitude and the phase shift for the echo with the time delay of $\tau_{i}$, respectively; *i* is the index of the target echo; and N(t) is the summation of the noise and the interference. The echo signal in an urban environment contains echoes from the background, the noise, and the interference. The power of the interference and the noise is significantly larger than that of the target echoes, as shown in Figure 1. The figure illustrates a scenario containing typical environmental characteristics during the detection. The background and the interference are still greater than 20 dB over the target echo after the MF processing and removing the background and the coherent interference. In order to be detectable, the target should be away from the strong interference.

It is difficult to detect the maneuvering target in urban environments due to the strong interference and the background noise. The background contains stationary objects, such as the buildings and the objects with periodic motion (e.g., trees swinging in the wind). The interference is intermittent and cannot be predicted. The echo from the target is usually very small (a SINR of less than −30 dB) and the Doppler information can easily be blurred in a strong noisy background in the frequency domain [5,8]. Hence, the target in Figure 1 cannot be detected effectively using the Doppler frequency estimation method or the TF analysis methods.

## 3. Target Detection Method

### 3.1. SMF Technique

It has been proved that the LFM signal produces optimal SNR using the matched filter for a special type of noise. The target cannot be detected effectively when the target echo is blurred by the strong noise or the interference [24,29,30,31,32,33,34,35]. However, the target may be detected when it is away from a strong interference area, although strong noise or interference can significantly reduce the detection probability. In such a case, the SINR of the echo signal should be improved using the priori information, such as transient and statistical characteristics of the noise and the interference, the gain differences of the matched filter over the target echo and the noise, etc.

The matched filter ensures that each output point has optimal SNR based on the original echoes. The effective length of the matched filter is limited in one pulse duration around the current point. The differences of the reflection coefficients of targets, as well as the strong noise or interference, cover the small target. As mentioned above, the gain of the matched filter over the target echo signal is almost constant that can be used to enhance the SINR for the small target echo. In this section, the self-adaption match filter (SMF) is proposed to reduce the power ratio between the big target echo (or the strong background and interference) and the small target echo.

The proposed self-adaption match filter uses the power gain information of the matched filter to improve the SINR of the small target echo obtained in urban environments. The SMF is given by:

(4) $$S_{mf}\left(t\right)=S_{r}\left(t\right)*F_{sa}\left(t\right),$$

(5) $$F_{sa}\left(t\right)=F\left(t\right)/G\left(t\right),$$

where ∗ represents the convolution operation, $S_{r}\left(t\right)$ is the echo of the transmitted LFM signal as in (3), $F\left(t\right)$ is the traditional matched filter, and the power factor $G\left(t\right)=\sup_{\tau \in [t-\frac{T_{p}}{2}\;\;t+\frac{T_{p}}{2}]}\left(\right|S_{r}\left(\tau \right)\left|\right)$ is the maximum amplitude of the filtered signal in the pulse duration $\left[t-T_{p}/2\;\;\;t+T_{p}/2\right]$. The output of the SMF is similar to the gain of the matched filter over echoes.

During the target detection process, the SNR of the echoes should be greater than a threshold for any analysis method [4,6,7]; otherwise, the performance of the detection system will be hard to improve. On the other side, it is difficult to improve the performance of the detection system when the SNR is good enough. The SMF can effectively improve the SINR of the target echo, keeping the SNR at the same level in a small range of the target echo under low SINR conditions compared with the MF, which will be validated in Section 4.1.

The power factor G(t) should have a lower threshold in order to ensure the SNR of the output of the matched filter for the small target echo. The power gain of the matched filter is difficult to control automatically when the echo is pure noise. The SNR of the target echo can deteriorate significantly if the small power factor is used. The threshold should be given considering the property of the transmitting signal, the target and the interference in the applications. Hence, priori knowledge of the target and the detection environment are necessary to ensure the performance of the SMF.

Note that the SMF ensures the optimal SNR for any output point. The power factor is used to automatically increase the SINR of the small target echo when the target is isolated from a source of strong noise.

### 3.2. ACA Method

In the target detection process, the noise and the interference are additive. The coherent background signal, including the echoes from the stationary targets and the coherent interference, can be effectively reduced using the repeated measurements. The quality of the echoes from the moving target can be significantly improved using matched filtering. In addition, the performance of the MF is almost unaffected during the background removal for the moving target. Considering the phase and the amplitude together, the echoes after the MF are not accumulated. Hence, the coherent accumulation for the echoes after the SMF (or MF) cannot be used.

However, the differences in the transient and the statistical properties between the target echo and the interference can be used to further improve the SINR of the echoes after the SMF. The phases of the echoes from the target vary due to the velocity of the target. The amplitudes are cumulative since the target positions are located in the same cell with high probability during the repeated tests within a limited period of time. However, the phases and the amplitudes of the residual noise and the interference are random and, therefore, not cumulative. The amplitude coherent accumulation is given by:

(6) $$S_{sum}\left(t\right)=\sum_{n}\left|S_{mf}^{n}\left(t\right)\right|,$$

where *n* is the index of the repeated tests, and |x| represents the absolute value of x.

**Figure 2:**
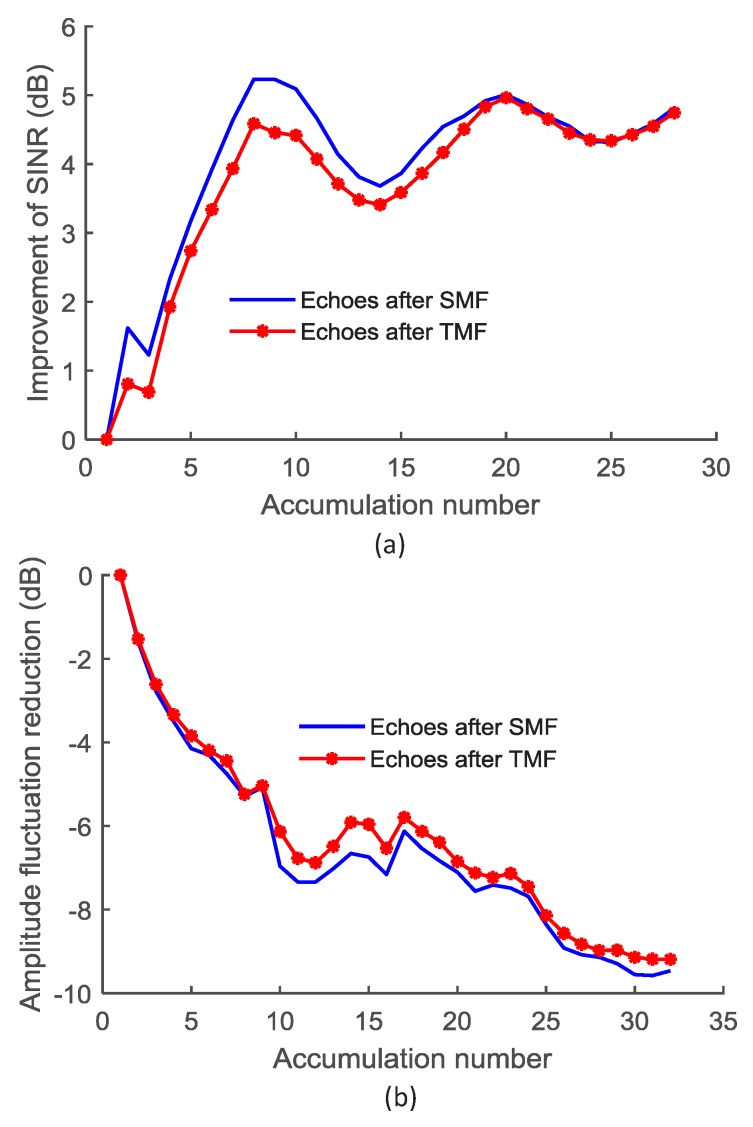
The performance of the ACA (Amplitude Coherence Accumulation) method for the echoes after processed by SMF (Self-adaption Matched Filter) and TMF (Traditional Matched Filter) , average result from 32 tests. (**a**) The SINR improvements with the accumulation number; (**b**) The amplitude fluctuation reduction of the noise/interference with the accumulation number. The frequency modulation rate of the LFM (Linear Frequency Modulation) signal is $\beta =6.35\times 10^{13}$, the bandwidth is BW=60 MHz, the sampling rate is 184.32 MHz, the frequency center is 1.83 GHz and the pulse repetition rate is 600 Hz. The pulse duration time is about 0.945 μs, i.e., 174 sampling points.

The amplitude accumulation gains of the test result between the moving target and the noises (including the random noise and the interference) are investigated. The echoes are processed using the SMF and the traditional matched filter (TMF), respectively and the results are shown in Figure 2. It can be seen from the figure that the SINR improvement and the amplitude fluctuation reduction of the noise/interference can be used to enhance the feature of the target echo. The SMF outperforms the TMF.

The SINR improvement is limited by the accumulation time using the ACA method for the moving target since the time positions of the amplitude peaks from different tests vary. The time delay intervals between the peaks depend on the velocity of the target. It can be seen from Figure 2a that the amplitudes of the target echoes are cumulative when the time delay interval is less than a limited time interval, that is, the accumulation gain increases with the increase of the accumulation number when the accumulation number is less than 8, which provides a reference value of coherent detection times in the target detection process. The result shown in Figure 2 is the average result of the data from 32 MonteCarlo experiments. The fluctuation of the SINR improvement is less than 0.9 dB. From the amplitude accumulation experiments we can see that an accumulation number of 8 provide the best improvement. The amplitude fluctuation of the noise/interference can be effectively reduced with the increase of the accumulation number as shown in Figure 2b. The fluctuation of the amplitude fluctuation reduction is less than 0.8 dB.

### 3.3. BDD Technique

**Figure 3:**
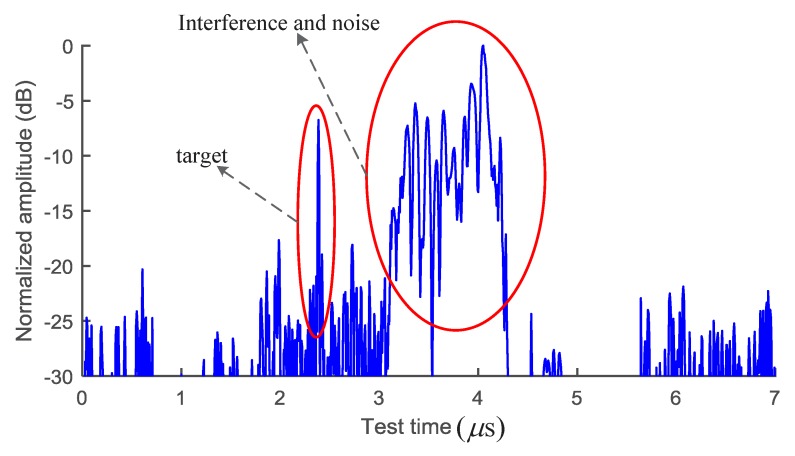
The echoes after SMF and ACA.

The amplitude of the target echoes after the SMF and the ACA processing may not be evaluated directly, although, the SINR can be improved significantly as shown in Figure 3. The data shown in Figure 3 is one snapshot in the data set used in the analysis for Figure 2. Nevertheless, the features of the target echoes after processed by either the SMF or ACA method become obvious. The target can be distinguished according to the following two factors: First, the SNR in the range around the target echo is good due to the proposed SMF and ACA method. Second, the fluctuation characteristics of the target echo and the nose/interference are different. The fluctuation feature of the target echo can be enhanced further using the time window function. According to the above two aspects, the BDD technique is proposed as:

(7) $$S_{d}\left(t\right)=\frac{S_{sum}\left(t\right)-S_{sum}\left(t-\Delta t\right)}{\Delta t}*\frac{S_{sum}\left(t\right)-S_{sum}\left(t+\Delta t\right)}{\Delta t},$$

where Δt is the time interval determined by the sampling interval and the bandwidth of the transmitting LFM signal.

The BDD approach depends on the SINR in a small range around the target echo and the slowly-varying property of the interference after the SMF and the ACA. The requirement of the SINR around the target echoes for the BDD technique is analyzed in Section 4. In summary, the proposed self-adaption moving target detection (SAMTD) approach consists of five steps as follows:

Step 1 Detect the target repetitively using a single-component LFM signal in a urban environment and remove the background noise using the priori information;
Step 2 Encode the echoes using the SMF and remove the coherent part based on the test data;
Step 3 Employ ACA method to improve the SINR and reduce the amplitude fluctuation of the interference;
Step 4 Enhance the differences of the target echo and the interference using the BDD technique;
Step 5 Obtain the target location by searching the peak position of the echo after post-processing.

## 4. Experiments and Analysis

In this section, the proposed detection approach is validated using simulations and experiments. During the experiments, the target is not in the strong interference area. The SINR is below −30 dB. The test data of a moving target are processed using the proposed SAMTD approach.

In the experiment, single base detection radar in the scanning mode is used. The LFM with frequency modulation rate of $\beta =6.35\times 10^{13}$ and bandwidth of BW=60 MHz are used. The pulse repetition rate is 600 Hz, the sampling rate is 184.32 MHz and the frequency center is 1.83 GHz. Two test scenarios, i.e., moving target in the urban environment and moving target in the open field are considered. The distance between the target and the radar is less than 10 km. At least eight repeated measurements for each test angle are performed in order to obtain sufficient data for the analysis.

### 4.1. SMF Technique

The SMF technique ensures the optimal SNR for each output point and enhances the SINR compared with the TMF when strong interference exists. The SINR can be improved for small targets that are isolated from strong interference. For a target close to strong interference, the SMF performs similar to the TMF.

**Figure 4:**
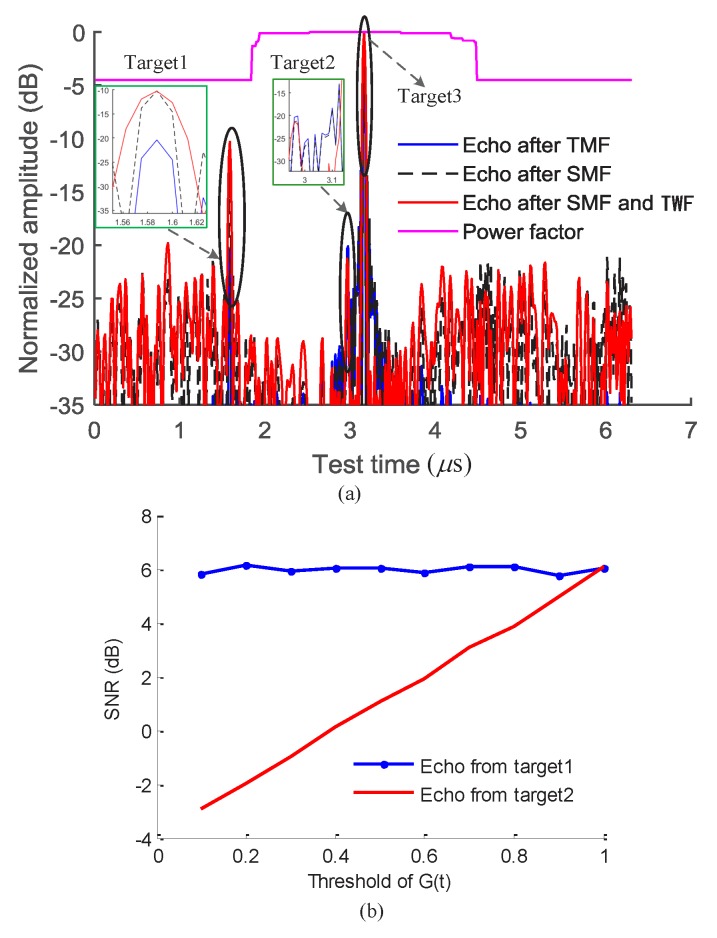
(**a**) Comparison of SMF and TMF; (**b**) The SNR of the small target echo corresponding to the threshold of the power factor.

Three simulated target echoes, with power ratios 1:1:100, are concluded in the Figure 4a. The threshold of the power factor is 0.6 in order to make the SNR of the echo from the target 2 positive according to the result shown in Figure 4b. The figure shows that the power of the small echo of target 1 (target 1 is away from the high-power echo area) can be improved using the SMF method. However, the SNR around the target echo cannot be improved as shown in Figure 4b. The performance of the SMF for the small target echo close to the high-power echo is similar to the performance of TMF, however, the SNR of the echo from the small target close to the strong echo is determined by the threshold of the power factor G(t). The time window function (TWF) can reduce the time side lobe of the target echoes [17]. Hence, the small target echo close to the high-power echo can be distinguished more easily when the TWF is used, as shown in Figure 4a.

### 4.2. ACA Method

Once the coherent parts of the echoes, i.e., the stationary target echo, the background, and the coherent interference, are removed, the remaining echoes are not cumulative. In order to improve the SINR using repeated measurements, the ACA method is proposed. The amplitudes of the target echoes are cumulative since the target located in the same sampling interval with high probability.

**Figure 5:**
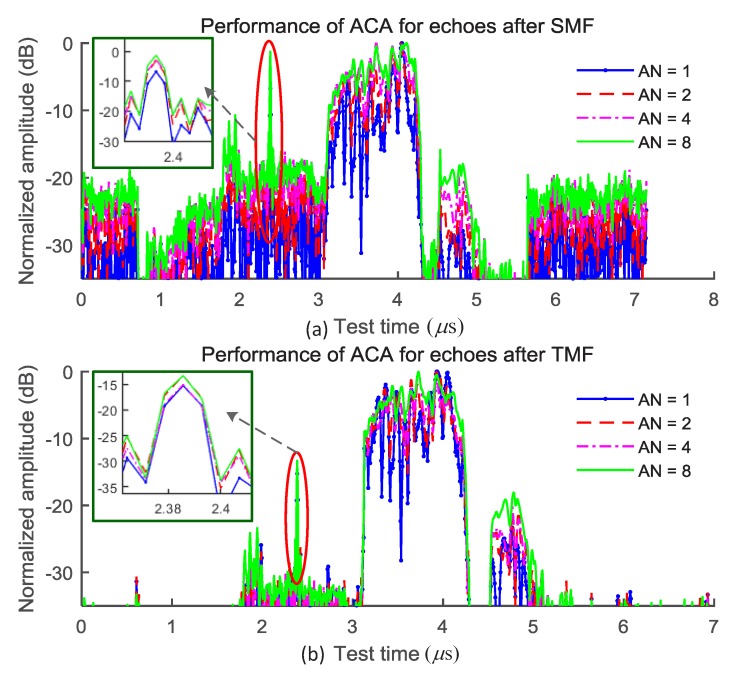
Performance comparison of the ACA method under different accumulation numbers. (**a**) The performance of the ACA based on the echoes filtered by SMF; (**b**) The performance of the ACA based on the echoes filtered by TMF.

In this section, the test data are analyzed experimentally since the distributions of noises and the interference are not known. Figure 5 shows the echoes after the MF processing and the removal of the background and the coherent part. The accumulation number (AN) is 1, 2, 4 and 8, respectively. It can be seen from the figure that the echoes after the SMF and the TMF can be improved, which is consistent with the results shown in Figure 2. However, the SINR of the output of the SMF is better than that of the TMF and correspondingly, the performance of the ACA method is better based on the echo filtered by the SMF.

### 4.3. BDD Technique

The SMF and the ACA methods improve the SINR of the echoes and reduce the amplitude fluctuation of the interference/noise, as described in Section 3.2 and Section 4.1, respectively. However, the power of the interference may still be greater than the power of the target echo, even though the accumulation number reaches 8, as shown in Figure 5a.

The BDD technique extracts the signature of the target echo based on the difference of the amplitude fluctuation between the target echoes and the interference. The BDD method depends on the good SNR in the target echo area and the reduced amplitude fluctuation of echoes in the strong interference area.

**Figure 6:**
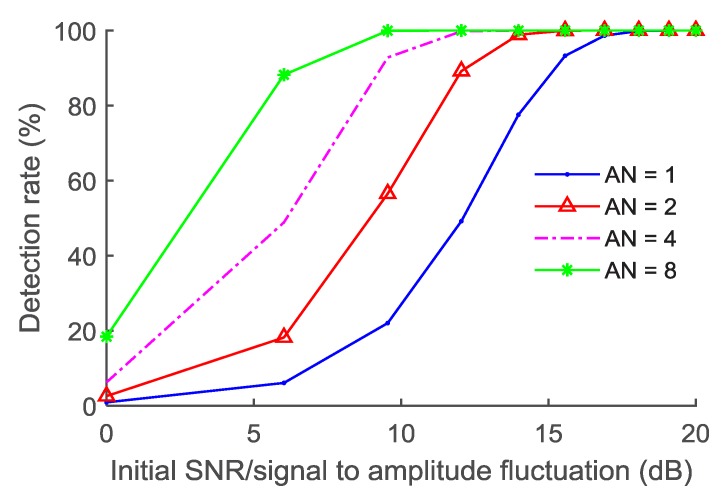
Performance of the BDD method under the different SNR of the processed echo.

Figure 6 shows the performance of the BDD technique corresponding to different accumulation numbers under different SNR (or amplitude fluctuations of the interference). The ACA method is ineffective when AN = 1. The SNR and the signal to amplitude fluctuation ratio are equivalent since the noise and the amplitude fluctuation approximately belong to the same distribution. It can be seen from the figure that the SNR should be higher than 16 dB in order to ensure the high performance of the BDD method using a single test.

### 4.4. Real Data Results

**Figure 7:**
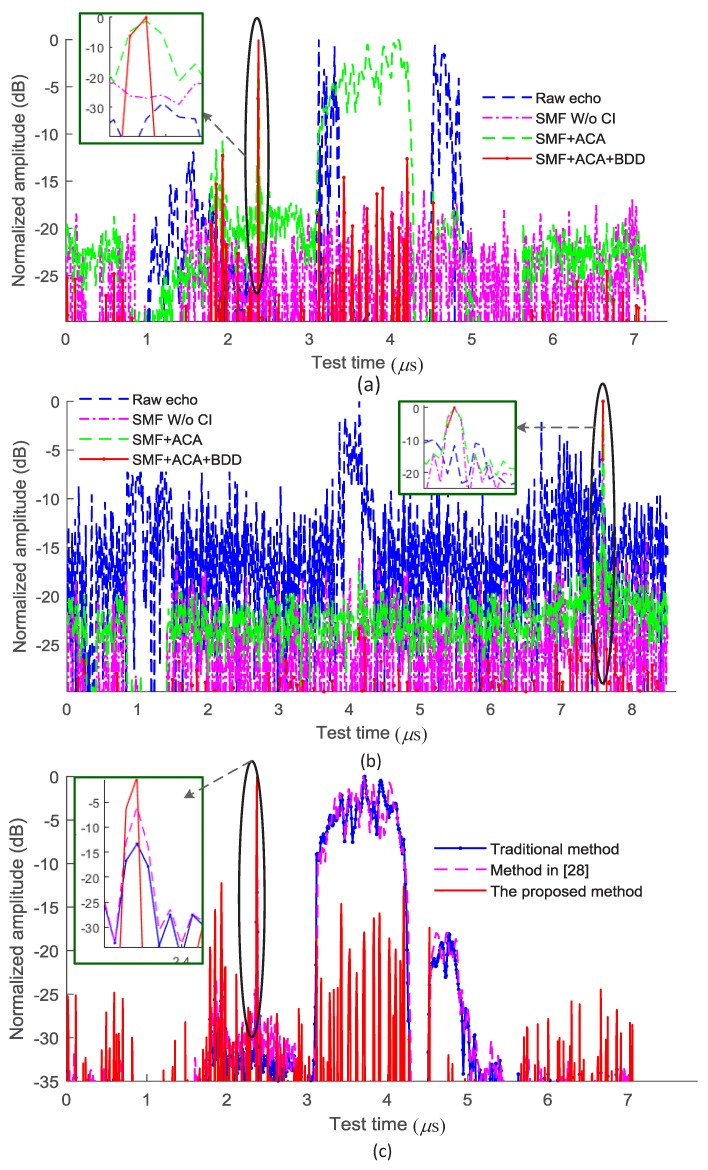
The performance of the proposed method based on the experimental data. (**a**) The result for Scenarios 1; (**b**) The result for Scenarios 2; (**c**) Comparison between the traditional method, the method in [28] and the proposed method for Scenario 1.

In order to validate the proposed method, experiments in two scenarios, i.e., moving target in the urban environment (Scenario 1) and moving target in the open field (Scenario 2), are conducted. The speed of the target is about 20 km/h. The setting of the test system is provided in Section 4.1. The processing results are shown in Figure 7. The detection rate shown in Figure 7 is obtained from the 100 tests in the environments with similar SNR. The SINR of the original echoes is about −30 dB in Scenario 1. The SNR for the original echoes is about −10 dB in Scenario 2. The ANs are 8 and 2 in Figure 7a,b, respectively. The SINR of the processed data after the SMF processing is improved to about −9 dB after removal of the background noise and the coherent interference (CI) in Scenario 1 and the detection rate is 96.7%, 100%, and 100% for AN = 1, 2, and 4, respectively. Figure 7b shows that the proposed method can work well in the high SINR test environments. Compared with the traditional target detection method and the method presented in [28], the proposed method can suppress high power interference effectively, and estimate the target position with high efficiency as illustrated in Figure 7c. In [28], the coherently integrated generalized cubic phase function, which improves the SNR through coherently integrating the generalized cubic phase function, is used to improve the quality of the target echoes.

Compared with the range Doppler method, the proposed method can detect small moving target in the low SINR test environment using a single test. The range Doppler method is hard to be used in Scenario 1 due to the low SINR, the uncontrollable target speed and low repetition rate. Furthermore, the TF analysis methods cannot be used in Scenario 1 due to the overlapped echoes in the low SINR test environment and low repetition rate.

## 5. Conclusions

In this paper, a self-adaption moving target detection method that consisted of the SMF, ACA, and BDD techniques was proposed. The SMF technique was presented to improve the SINR of the small moving target by using the power factor. The power factor was generated according to the power distribution of the echoes. The SINR of the target echo could be improved using the amplitude information within proper test time based on multiple measurements. The ACA technique enhanced the difference of the statistical and transient characteristics between the target echo and the interference. Finally, the BDD technique was proposed to distinguish the target more easily. The simulations and experiments have demonstrated and validated that the proposed method can detect the small moving target with high probability using multiple test data in low SINR environment.

## Abbreviations

The following abbreviations are used in this manuscript:

ACA	Amplitude Coherence Accumulation
AN	Accumulation Number
BDD	Bi-Directional Difference
CI	Coherent Interference
CPF	Cubic Phase Function
LFM	Linear Frequency Modulation
MF	Matched Filter
SAMTD	Self-Adaption Moving Target Detection
SINR	Signal-to-Interference-Noise Ratio
SMF	Self-adaption Matched Filter
STFT	Short-Time Fourier Transform
TF	Time-Frequency
TMF	Traditional Matched Filter
TSL	Time Side Lobe
TWF	Time Window Function
WT	Wavelet Transform
WVD	Wigner–Ville Distribution
